# Probing the Role of *Meso*‐DAP and Lysine for Growth and Pathogenicity of *Pseudomonas aeruginosa*

**DOI:** 10.1002/mbo3.70200

**Published:** 2025-12-22

**Authors:** Daniel A. Hawkins, Rachael E. Impey, Charlotte K. Hind, J. Mark Sutton, Tatiana P. Soares da Costa

**Affiliations:** ^1^ School of Agriculture, Food and Wine, Waite Research Institute The University of Adelaide, Waite Campus Glen Osmond South Australia Australia; ^2^ Department of Biochemistry and Chemistry, La Trobe Institute for Molecular Science La Trobe University Bundoora Victoria Australia; ^3^ Antimicrobial Discovery, Development and Diagnostics (AD^3^), United Kingdom Health Security Agency (UKHSA), Porton Down Salisbury Wiltshire UK

**Keywords:** amino acids, antibiotic resistance, diaminopimelate pathway, gene deletion, lysine, *Pseudomonas aeruginosa*

## Abstract

Antibiotic‐resistant bacteria represent a major global challenge as increasing infections become recalcitrant to standard treatments. A lack of novel therapeutics entering the market in the past 30 years further exacerbates this issue and highlights the importance of identifying and validating novel antibiotic targets. In this study, we explored prospective therapeutic targets by examining two metabolites in the lysine biosynthesis pathway, *meso*‐diaminopimelate (DAP) and lysine, within the critically listed pathogen *Pseudomonas aeruginosa*. These metabolites are involved in bacterial cell wall and protein synthesis; therefore, enzymes present in this pathway represent potential targets for novel therapeutics. To elucidate the validity of these targets, we generated for the first time, gene deletion mutants of the *P. aeruginosa* DHDPR‐ and DAPDC‐encoding genes using a two‐step allelic exchange method. Both the mutants resulted in a lethal phenotype that could be rescued by supplementation with *meso*‐DAP and/or lysine. We subsequently characterized the mutants' pathogenicity in a *Galleria mellonella* infection model. The DHDPR mutant was unable to provide a lethal infection in this model. Given the importance of these metabolites to membrane and cell wall synthesis, we investigated membrane permeability utilizing a fluorescent probe assay and transmission electron microscopy. Due to their increased membrane permeability, these mutants exhibited greater sensitivity to antibiotics commonly used against *Pseudomonas* infections. Overall, this study highlights that targeting the lysine biosynthesis pathway could enhance bacterial susceptibility to existing antibiotics, supporting its development as an adjuvant strategy to potentiate current treatments and extend their clinical utility.

## 1 Introduction

The well‐documented rise of antibiotic‐resistant bacteria combined with a dwindling antibiotic discovery pipeline is threatening our ability to treat common bacterial infections. According to the World Health Organization (WHO), one of the highest priority pathogens is *Pseudomonas aeruginosa*, which is a leading cause of mortality in people with cystic fibrosis and is commonly associated with nosocomial infections (Emerson et al. 2002; Tacconelli et al. 2018). Both intrinsic and acquired resistance mechanisms contribute to the pathogenicity of *P. aeruginosa* that, in turn, has led to an increase in reports of extremely drug‐resistant strains for which there is little to no currently available treatments (Estahbanati et al. 2002; Horcajada et al. 2019; Palavutitotai et al. 2018; Impey et al. 2020). Thus, the identification and characterization of novel antibiotic targets in this pathogen is of utmost importance to tackle the global antibiotic resistance crisis.

An area of interest that is yet to yield clinically available antibiotics is centered around targeting the building blocks of proteins, amino acids. Bacteria have the ability to synthesize all 20 amino acids *de novo*, including the nine essential amino acids that animals cannot produce and must obtain through their diet (Idrees et al. 2020). Indeed, several studies have focused on inhibiting enzymes in the pathways leading to the synthesis of essential amino acids, such as branched‐chain and aromatic amino acids (Grandoni et al. 1998; Impey and Soares da Costa 2018; Amorim Franco and Blanchard 2017; Parish and Stoker 2002). Enzymes in these pathways have been shown to be essential for bacterial growth and survival, and the “druggability” of each prospective target has been elucidated through structural and functional studies (Amorim Franco and Blanchard 2017).

**Figure 1:**
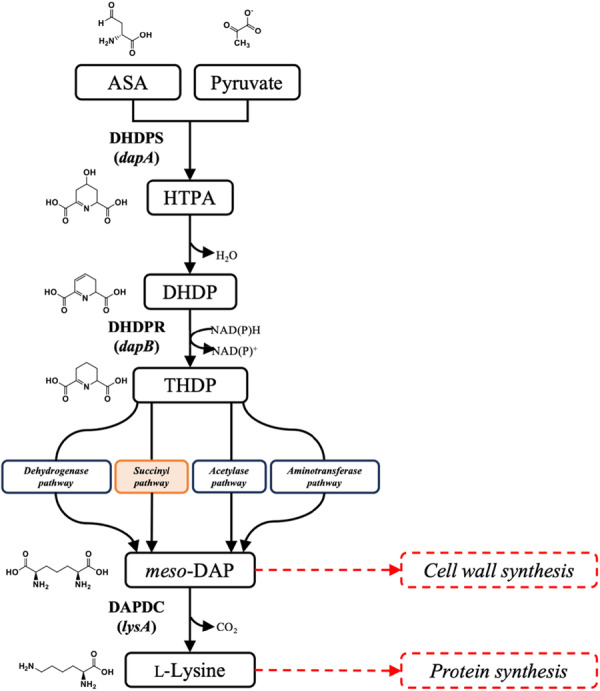
Diaminopimelate (DAP) pathway in *Pseudomonas aeruginosa*. The DAP pathway begins with the condensation of ASA and pyruvate to HTPA facilitated by the enzyme, DHDPS. HTPA is non‐enzymatically dehydrated to DHDP. DHDP is further reduced to THDP by DHDPR. The pathway then diverges to one of four sub‐pathways, dependent on the organism, before reconverging at the production of *meso*‐DAP. *Pseudomonas aeruginosa* utilizes the succinyl sub‐pathway (orange). *meso‐*DAP can be utilized in the crosslinking of the Gram‐negative bacterial peptidoglycan layer, a target for antibiotics such as β‐lactams. The decarboxylation of *meso*‐DAP by DAPDC produces lysine, which is utilized for protein synthesis.

An essential amino acid biosynthesis pathway that remains relatively unexplored is the diaminopimelate (DAP) pathway involved in lysine production (Figure 1). The pathway is also responsible for the production of *meso*‐DAP, which is utilized by Gram‐negative bacteria, including *P. aeruginosa*, for crosslinking of the cell wall (Hutton et al. 2007). The pathway begins with the condensation of pyruvate and aspartate semi‐aldehyde (ASA) to 4‐hydroxy‐2,3,4,5‐tetrahydrodipicolinic acid (HTPA), which is catalyzed by dihydrodipicolinate synthase (DHDPS, E.C. **4.3.3.7**) (Impey and Soares da Costa 2018; Hutton et al. 2007; Christoff et al. 2019; Soares da Costa et al. 2010). Following this, the dehydrated product, dihydrodipicolinate (DHDP), is reduced to 2,3,4,5‐tetrahydrodipicolinate in a NAD(P)H‐dependent reaction by dihydrodipicolinate reductase (DHDPR, E.C. **1.17.1.8**) (Devenish et al. 2010; Soares da Costa et al. 2015). The pathway then diverges into four sub‐pathways in a species‐specific manner (Hutton et al. 2007; Peverelli et al. 2016; Dogovski et al. 2012; Gupta et al. 2018). In *P. aeruginosa*, the succinyl pathway is utilized to produce *meso*‐DAP. Finally, *meso*‐DAP is converted to l‐lysine (hereinafter referred to as lysine) by diaminopimelate decarboxylase (DAPDC, E.C. **4.1.1.20**) (Peverelli et al. 2016; Dogovski et al. 2012). Given that the synthesis of bacterial cell wall and proteins are targets of current antibiotics, including ß‐lactams and aminoglycosides (Fernandes et al. 2013; Kotra et al. 2000), we propose that the inhibition of the DAP pathway could represent a novel strategy for antibiotic discovery as it would interfere with these two essential processes concurrently (Hutton et al. 2007).

Prior to commencing inhibitor discovery studies against various enzymes in this pathway, this project set out to assess the importance of *meso*‐DAP and lysine for the growth and pathogenicity of *P. aeruginosa*. This study aimed to generate *meso*‐DAP and lysine auxotrophs that would be devoid of DHDPR or DAPDC activity due to the deletion of their associated genes, *dapB* and *lysA*, respectively. Gene deletions in *P. aeruginosa* have historically been challenging to perform, with most published methods relying on plasmids with a ColE1 origin of replication, which replicates in *Escherichia coli* but not in *Pseudomonas* species (Hmelo et al. 2015). This study employed a seamless allelic exchange method to achieve this in *P. aeruginosa*. We subsequently performed supplementation experiments with the knockout strains using different concentrations of either *meso‐*DAP or lysine to assess their requirement for bacterial growth. We then elucidated the role of these metabolites on the pathogenicity of *P. aeruginosa* by using an *in vivo* infection model, *Galleria mellonella*. The effects of *meso*‐DAP and lysine depletion on membrane permeability and antibiotic susceptibility were also investigated for these generated mutants.

## 2 Results

### 2.1 Generation of *ΔdapB* and *ΔlysA* Gene Deletion Mutants

**Figure 2:**
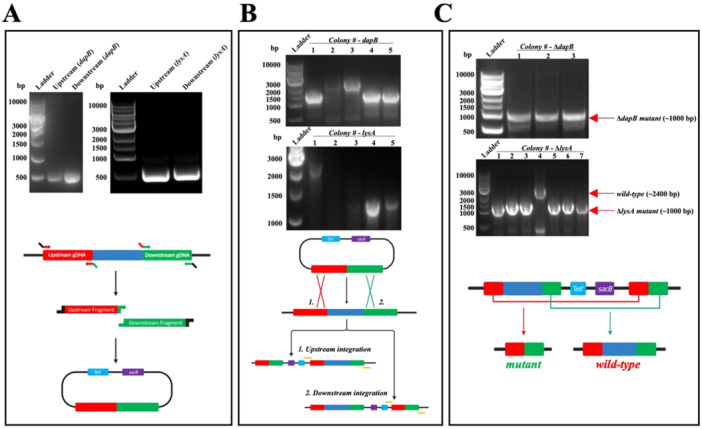
Generation of *dapB* and *lysA* gene deletions in *Pseudomonas aeruginosa*. (A) Amplification of the 500 bp genomic DNA regions that flank upstream and downstream of the *dapA* (left) or *lysA* (right) gene for ligation into the pEX18Tc plasmid. (B) Colony PCR screening of five transformant colonies (1–5) showing successful integration of the assembled “suicide vector” into the genome of *P. aeruginosa*. Colonies integrated downstream of either the *dapB* or *lysA* gene are shown by the larger PCR product (~ 2500 bp), whereas upstream integrations are shown by the smaller product (~ 1250 bp). (C) Representative colony PCR screening of three Δ*dapB* mutants and seven Δ*lysA* mutants. Colonies were selected for by tryptone, yeast and sucrose media. The ~1000 bp product indicates a successful *dapB* or *lysA* gene deletion, compared to the WT revertant colonies at ~2400 bp.

A two‐step allelic exchange method was utilized to generate a *dapB* and *lysA* knockout mutant in *P. aeruginosa* (PAO1) (Hmelo et al. 2015). This method requires two recombination events to occur, with the first event facilitated by the assembly of the “suicide vector.” To assemble the construct, the upstream and downstream regions that flank either *dapB* or *lysA* (500 bp) were amplified from the PAO1 genome (Figure 2A). Subsequently, these regions were ligated into digested pEX18Tc plasmid. The completed “suicide vector” construct was transformed into PAO1 using an optimized electroporation protocol (Huang and Wilks 2017). Positive transformants were screened for successful integration into the PAO1 genome via colony PCR (Figure 2B). From the five colonies screened for each suicide vector construct, either an upstream or downstream integration was observed. Colonies containing the *dapB* construct yielded three upstream (~ 1250 bp) and two downstream (~ 2500 bp) integrations (Figure 2B). Furthermore, the *lysA* construct was successfully integrated upstream (~ 1250 bp) in two colonies and integrated downstream (~ 2500 bp) in one colony (Figure 2B). The second homologous recombination event was initiated by plating the bacteria on TYS media, thus inducing *sacB* toxicity to the bacteria‐containing plasmids that have the suicide vector still integrated into the genome. To account for the potential knockout of either *dapB* or *lysA*, the media was supplemented with DAP and/or lysine at 1 mM final concentration. Colonies of this second recombination event were screened via colony PCR, which revealed six successful Δ*dapB* mutants out of 60 colonies examined and 10 successful Δ*lysA* mutants out of 29 colonies screened. Representative data of successful Δ*dapB* and Δ*lysA* mutants are shown, with knockout generation at ~1000 bp and the WT revertant seen by the larger band present on the agarose gel (Figure 2C). The 10% success rate for Δ*dapB* and 34% for Δ*lysA* is lower than that reported in the protocol papers (Hmelo et al. 2015; Huang and Wilks 2017), which may be attributed to the deletion of the *dapB* and Δ*lysA* gene being selected against, despite the supplementation. As expected, when the second counterselection was performed on non‐supplemented media, no successful mutants were obtained when similar numbers of bacteria were screened (data not shown). This indicates that the supplementation of the media is a critical step to allow for the assessment of a knockout of these potentially essential genes.

### 2.2 Effect of *Meso*‐DAP and Lysine Depletion on *P. aeruginosa* Growth

**Figure 3:**
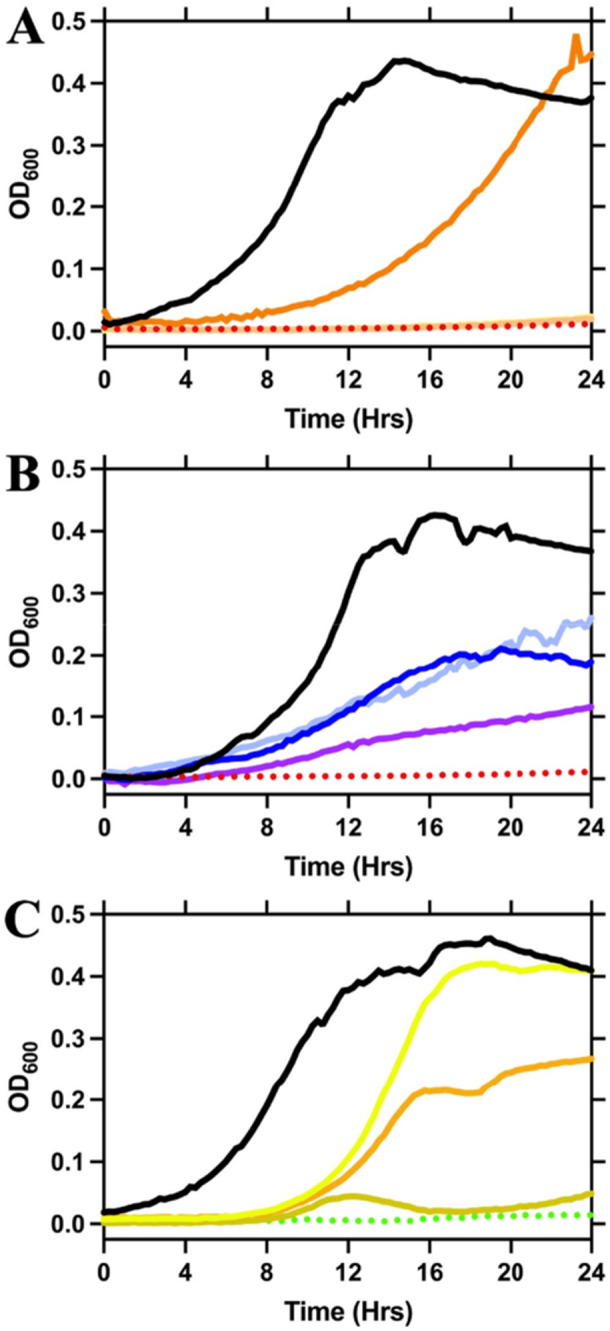
Phenotypic growth characterization of Δ*dapB* or Δ*lysA* mutant with and without supplementation. A 24‐h growth curve of *wild‐type* (black) compared against (A) Δ*dapB* mutant (red; dotted); Δ*dapB* supplemented with 1 mM (orange), 200 μM (light orange) and 40 μM (yellow) DAP; (B) Δ*dapB* mutant (red; dotted); Δ*dapB* supplemented with 1 mM (dark blue), 200 μM (light blue) and 40 μM (purple) lysine; (C) Δ*lysA* mutant (green; dotted); Δ*lysA* supplemented with 1 mM (yellow), 200 μM (orange) and 40 μM (gold) lysine. Bacteria were grown in MOPS minimal media with growth assessed by optical density at 600 nm (OD_600_). Results shown are typical of at least two independent biological experiments (*N* = 2).

To assess the impact of arresting *meso*‐DAP and lysine biosynthesis in the *dapB* and *lysA* knockout mutants, growth curve assays were performed, comparing both mutants against the WT PAO1 strain. The assays were completed over a 24‐h period, whereby the change in absorbance (OD_600_) was measured in 15 min intervals (Figure 3). The WT strain showed a typical growth profile with a plateau occurring at ~16 h (Figure 3A–C). The flat growth line for the Δ*dapB* mutant indicates that without supplementation, the bacteria are unable to grow (Figure 3A and B). Supplementation first with DAP was investigated at different concentrations (1 mM–40 µM), with a concentration of 1 mM providing rescued growth, albeit with a slight growth delay compared to WT (Figure 3A). Supplementation of the Δ*dapB* mutant with lysine (1 mM–40 µM) also rescued growth, although the gradient of the curve was not as steep, indicating a slower exponential phase compared to the WT (Figure 3B). Furthermore, lower concentrations of lysine seemed to rescue growth in a more dose‐dependent manner as opposed to the DAP supplementation (Figure 3B). In comparison, the flatline observed for the *lysA* knockout mutant indicates that the bacteria are unable to grow when lysine is absent in the cell or in the environment (Figure 3C). The supplementation of lysine (1 mM–40 µM) to the growth medium rescued growth of the Δ*lysA* mutant in a dose‐dependent manner, albeit growth was attenuated and slowed with the exponential phase occurring at approximately 12 h, whereas this is observed from the outset of the assay in the WT strain (Figure 3C). These studies highlight the conditional essentiality of the *dapB* and *lysA* genes, that is, *dapB and lysA,* are essential unless there is an exogenous source of *meso*‐DAP and/or lysine. Accordingly, we next set out to assess the ability of both Δ*dapB* and Δ*lysA* mutants to scavenge *meso*‐DAP and lysine from media to ascertain whether the mutations caused bacterial cell death or induced dormancy.

To investigate this, the Δ*dapB* and Δ*lysA* mutants were cultured either with or without DAP/lysine supplementation and grown overnight at 37°C. The following day, growth of each of the cultures was noted and secondary cultures were made by subculturing the original overnight into fresh minimal media with or without DAP/lysine supplementation as per Table 1. For Δ*dapB*, the initial culture did not grow as expected. However, when subcultured into either DAP or lysine containing media, growth was observed, indicating viable bacteria remained in the original culture, even though no growth was observed (Table 1). The same result was observed for the Δ*lysA* mutant, further supporting the premise that mutations in these genes do not cause cell death but rather led to a bacteriostatic effect. When the Δ*dapB* and Δ*lysA* mutants were initially grown with supplementation, and then the supplementation was taken away upon subculturing, no growth was observed (Table 1). This suggests that a constant exogenous source of supplementation is required for the growth of these bacterial mutants, although they remain viable regardless of growth conditions. We next sought to unravel whether these mutants were still pathogenic in an *in vivo* infection model.

**Table 1:** The effect of supplementation on Δ*dapB* and Δ*lysA* mutant growth in MOPS minimal media. Cultures grown for 20 h at 37°C. ( + ) is indicative of growth, (−) refers to no growth observed.

	Starter culture (Day 1)		Secondary culture (Day 2)	
	Supplementation	Growth (+/−)	Supplementation	Growth (+/−)
*ΔdapB*	none	—	1 mM DAP	+
	none	—	1 mM lysine	+
	1 mM DAP	+	none	—
	1 mM lysine	+	none	—
Δ*lysA*	none	—	1 mM lysine	+
	1 mM lysine	+	none	—

### 2.3 Effect of *Meso*‐DAP and Lysine Depletion on *P. aeruginosa* Pathogenicity

**Figure 4:**
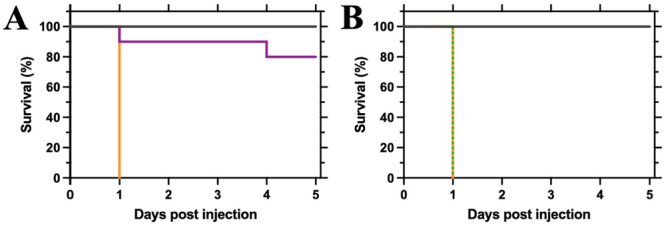
Pathogenicity of Δ*dapB* or Δ*lysA* mutant in a *Galleria mellonella* infection model. *G. mellonella* populations were subjected to infection (OD_600_ = 1 × 10^6^) via injection and monitored for 5 days. (A) Δ*dapB* mutant infection (purple; solid) compared to *wild‐type* (orange; solid), *P* = < 0.0001, Log‐rank (Mantel‐Cox) test. (B) Δ*lysA* mutant infection (green; dashed) compared to *wild‐type* (orange; dashed), *P* = > 0.9999, Log‐rank (Mantel‐Cox) test. A PBS control (black; solid) was also included for both experiments. Each treatment group contained 10 larvae (*N* = 10).

To gain an understanding of the role that *meso*‐DAP and lysine plays in the pathogenicity of *P. aeruginosa*, a *G. mellonella* infection model was utilized to compare the mutant strains to the WT. The *G. mellonella* model is a well**‐**established model to assess the pathogenicity of bacterial strains given the similarities of their innate immune response to mammals (Benthall et al. 2015). Cultures of WT, Δ*dapB,* or Δ*lysA* were grown overnight in rich media and then diluted in PBS to an OD_600_ of 1 × 10^−^^6^ (2 CFU/10 μL). *G. mellonella* populations (*N* = 10) were subjected to either WT, Δ*dapB,* or Δ*lysA* infection and monitored for 5 days. Infection with WT PAO1 resulted in lethality of the entire population after 24 h (Figure 4A,B). When *G. mellonella* was challenged with a Δ*dapB* infection, a significant reduction in pathogenicity was observed, with an 80% survival rate after 5 days (*P* = < 0.0001, Figure 4A), highlighting the essentiality of *meso*‐DAP for the virulence of the strain in this *G. mellonella* model. Although addition of lysine to minimal media can rescue growth of this mutant in the previously reported growth curve analysis, this mutant cannot proliferate and grow effectively in this model to cause sufficient lethality. In contrast, a Δ*lysA* infection was still able to grow and proliferate, resulting in lethality of the entire *G. mellonella* population after 24 h (Figure 4B), similarly to what was observed with the WT strain. Therefore, although growth is severely affected and disrupted with the loss of the *lysA* gene, it would appear as though *P. aeruginosa* can scavenge and sequester lysine from *G. mellonella* and restore its pathogenicity.

### 2.4 Effect of *Meso*‐DAP and Lysine Depletion on *P. aeruginosa* Membrane Permeability

**Figure 5:**
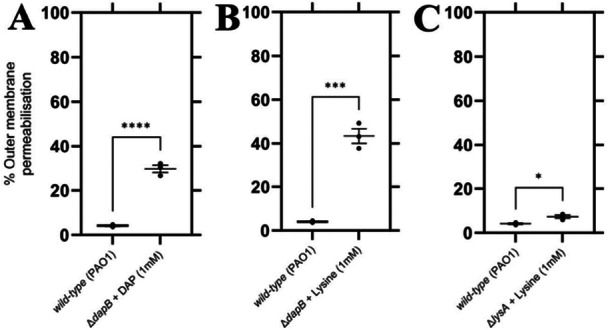
Membrane permeability assessment of Δ*dapB* or Δ*lysA* mutant in comparison to *wild‐type* (PAO1) *P. aeruginosa*. NPN membrane permeability assay was performed over 20 min by measuring fluorescence intensity (FI) in 1.5 min intervals. Comparison of normalized outer membrane permeabilization percentage of *wild‐type P. aeruginosa* versus (A) Δ*dapB* + 1 mM DAP, *P* = < 0.0001, unpaired Student's two‐tailed *t*‐test; (B) Δ*dapB* + 1 mM lysine, *p* = 0.0003, unpaired Student's two‐tailed *t*‐test; and (C) Δ*lysA* + 1 mM lysine, *p* = 0.0137, unpaired Student's two‐tailed *t*‐test. Error bars represent mean ± S.E.M. Results shown are of three independent biological experiments (*N* = 3). *p ≤ 0.05, ***p ˂ 0.001, ****p < 0.0001.

Given the importance of *meso*‐DAP in bacterial cell wall biosynthesis, we set out to investigate whether *meso*‐DAP and lysine depletion would result in changes in membrane permeability. To do this, we used *N*‐phenylnaphtylamine (NPN) as a fluorescent probe (Helander and Mattila‐Sandholm 2000). When the bacterial membrane is intact, hydrophobic substances such as NPN cannot penetrate. However, once the membrane is compromised, NPN is able to bind to the internal phospholipid layer, which then provides a fluorescent signal (Helander and Mattila‐Sandholm 2000). For Δ*dapB* mutants supplemented with either DAP or lysine, outer membrane permeabilization increased by an average of 25.5% and 39.3%, respectively (*p* = < 0.0001, *p* = 0.0003; Figure 5A,B). The increase indicates the membrane permeability in this strain is enhanced, highlighting the important role *meso*‐DAP plays in strengthening the cell membrane. The Δ*lysA* mutant resulted in a smaller, albeit statistically significant increase of 3.19% in outer membrane permeabilization (*p* = 0.0137; Figure 5C).

**Figure 6:**
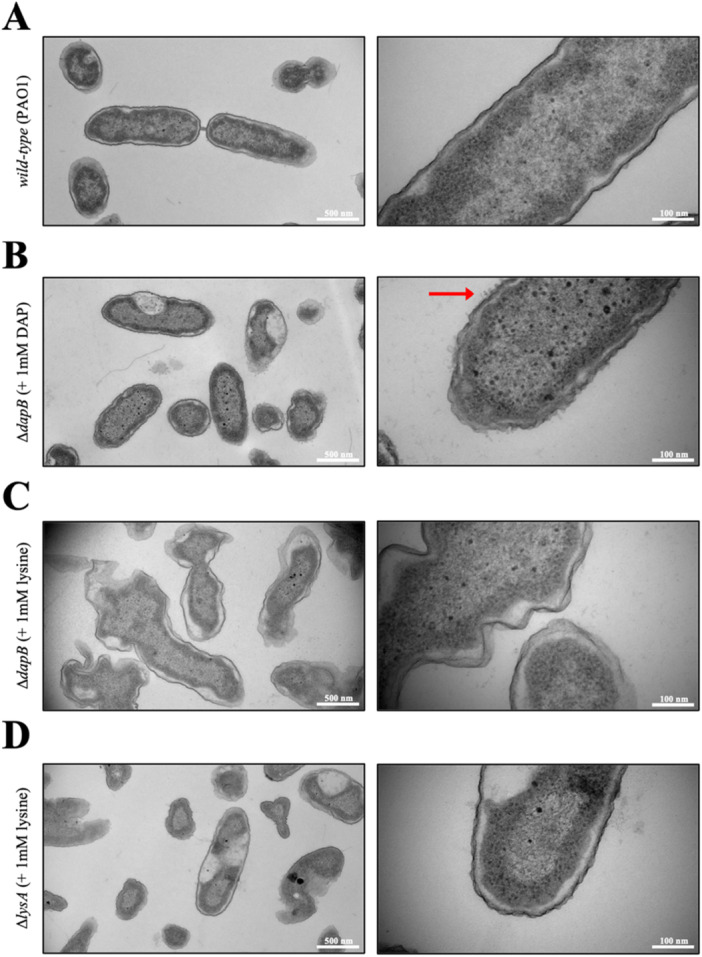
Transmission electron micrographs of *Pseudomonas aeruginosa*. (A) *Wild‐type P. aeruginosa* PAO1, (B) Δ*dapB* mutant ( + 1 mM DAP); small membrane protrusions indicated by red arrow, (C) Δ*dapB* mutant ( + 1 mM lysine) and (D) Δ*lysA* mutant ( + 1 mM lysine). Left panels: 13,000 × magnification, scale bars are equal to 500 nm; right panels: 49,000 × magnification, scale bars are equal to 100 nm.

To determine if there were any morphological changes in the membrane, transmission electron microscopy (TEM) was employed (Figure 6). WT *P. aeruginosa* exhibited the expected rod‐shaped morphology with defined outer membrane borders (Figure 6A). The Δ*dapB* mutant that had been supplemented with DAP showed the stereotypical rod‐shaped morphology like the WT; however, upon close inspection, small protrusions from the outer membrane were observed, potentially indicative of a weakened membrane (Figure 6B; red arrow). Moreover, the lysine supplemented Δ*dapB* mutant exhibited strikingly different overall morphology compared to the WT. The non‐defined shapes of this mutant strain suggest that presence of DAP plays an important role in overall cell morphology and thus membrane strength, which would be expected (Figure 6C). Therefore, although lysine can rescue growth of the Δ*dapB* mutant, by subverting the necessity of the lysine biosynthesis pathway, the mutant's membrane permeability and morphology are compromised due to the lack of DAP being available. The Δ*lysA* mutant showed varying shapes with some cells having incomplete membranes, although this was only apparent on a subset of the population surveyed (Figure 6D). The observed visual changes in the outer membrane structure further suggests that the depletion of DAP and lysine results in increased membrane permeability that could be exploited for the development of novel antibiotics or adjuvants that could help re‐sensitize resistant populations to current antibiotics.

### 2.5 Susceptibility of *ΔdapB* and *ΔlysA* Gene Deletion Mutants to Antibiotics

We next sought to understand whether the mutations harbored by the generated strains would have an increase in their antibiotic susceptibility, compared to the WT strain. Antibacterial assays were set up to establish minimum inhibitory concentration (MIC) values for the strains against antibiotics commonly used against *P. aeruginosa* infections, namely, ciprofloxacin and tobramycin (Table 2). The Δ*dapB* mutant supplemented with 1 mM DAP showed increased susceptibility to both antibiotics tested (Table 2). An increased susceptibility was seen against ciprofloxacin with an MIC value of 1–4 μg/mL compared to 4–8 μg/mL for the WT (Table 2). Increased susceptibility against tobramycin was also seen for this mutant with an MIC value of 0.5 μg/mL compared to 1–2 μg/mL for the WT strain (Table 2). The lysine supplemented Δ*dapB* mutant showed an increased susceptibility to ciprofloxacin and tobramycin with an MIC value of 0.5 μg/mL for both antibiotics (Table 2). The MIC range for the Δ*lysA* mutant against the two antibiotics tested was in range of the WT MIC for both ciprofloxacin and tobramycin (Table 2). The findings suggest that the Δ*dapB* gene deletion increases susceptibility to ciprofloxacin and tobramycin, particularly when supplemented with DAP or lysine, indicating a potential vulnerability that could be exploited for enhancing antibiotic efficacy. These results highlight the potential of manipulating the DAP pathway to increase bacterial susceptibility to existing antibiotics, offering insight for future therapeutic strategies.

**Table 2:** Antibacterial assays. Minimum inhibitory concentration (MIC) values for *wild‐type* PAO1, Δ*dapB*, and Δ*lysA* against ciprofloxacin and tobramycin (*N* = 3).

	MIC range (µg/mL)	
	Ciprofloxacin	Tobramycin
*Wild‐type* (PAO1)	4–8	1–2
Δ*dapB* (+1 mM DAP)	1–4	0.5
Δ*dapB* (+1 mM lysine)	0.5	0.5
Δ*lysA* (+200 µM lysine)	4	0.5

## 3 Discussion

There is a pressing need to identify and validate novel antibiotic targets to address the ominous rise of antibiotic‐resistant bacteria that is threatening our global healthcare systems. This is particularly pertinent for high priority Gram‐negative bacteria like *P. aeruginosa*, for which effective treatments are rapidly diminishing (Ibrahim et al. 2020). Investigating novel pathways, such as the DAP pathway, for antibiotic development requires understanding of the role of the resulting metabolites, namely, *meso*‐DAP and lysine. Although the role of *meso*‐DAP and lysine have been reported, we sought to understand the phenotypic effect of depleted strains in *P. aeruginosa* by manipulating the *dapB* or the *lysA* gene, and thus, their associated enzymes. As many antibiotic targets are the products of essential genes, genome‐wide studies have attempted to identify possible targets in *P. aeruginosa*. These studies often utilize transposon libraries, which are generated via the insertion of transposon DNA into the bacterial genome to disrupt normal gene function (Jacobs et al. 2003). Gene essentiality is then determined either by the frequency of transposon insertion or via assessment of growth defects for each individual transposon mutant (Liberati et al. 2006; Lee et al. 2015; Poulsen et al. 2019; Skurnik et al. 2013). However, such approaches come with limitations. Determining essentiality by the absence or low frequency of transposon insertions is heavily influenced by the breadth of the library itself (Chao et al. 2016). Furthermore, assessing growth defects relies on the constitution of the media, which could result in the mutant being inadvertently supplemented, if not properly considered (Chao et al. 2016).

Utilizing an allelic exchange method (Hmelo et al. 2015; Huang and Wilks 2017), we were able to generate Δ*dapB* and Δ*lysA* gene deletion mutants in *P. aeruginosa* for the first time. The generation of a seamless knockout allows us to overcome the limitation of relying upon a transposon insertion into the *dapB* or *lysA* gene to determine the effect of depleting either *meso*‐DAP or lysine production, and thus, helps define the essentiality of the genes. We investigated the growth phenotype of the Δ*dapB* and Δ*lysA* mutants in MOPS minimal media, with the controlled addition of DAP and/or lysine to avoid any unintentional supplementation within the media. For the Δ*dapB* and Δ*lysA* mutants, no growth was observed unless DAP or lysine was added. Interestingly, the Δ*dapB* could be rescued by lysine alone, indicating that the pathway can be bypassed, suggesting that *meso*‐DAP is not a requirement for growth. We hypothesize that in a *meso*‐DAP deficient mutant in *P. aeruginosa*, lysine could be utilized in the cell wall in place of *meso*‐DAP. This is observed in Gram‐positive bacteria, where lysine is used in the cell wall instead of *meso*‐DAP (Ruane et al. 2013). However, at this point, it was uncertain as to whether the lack of *meso*‐DAP in the lysine supplemented strain would incur a fitness penalty. Moreover, the ability to supplement the strains with the depleted metabolites gave an indication that the impaired growth phenotype was due to the loss of the *dapB* or *lysA* gene. Although previous studies have proposed that this method of allelic exchange could not be used to screen for essential genes (Hmelo et al. 2015), we have shown that this is possible by using supplementation during and after the generation of the gene deletion mutants, as long as there is a suitable metabolite that can be sourced to rescue growth and viability.

We further investigated whether disruption of the *dapB* and *lysA* genes caused cell death or simply inhibited growth. Cultures devoid of any supplementation were shown not to grow, however when a subculture was established containing supplement, growth was able to resume. This suggests that the depletion of DAP and lysine in these strains causes a severe fitness penalty, although they are still viable. In this instance, the mutant strains present as viable but non‐culturable cells when an exogenous supply of DAP and/or lysine is absent (Kaprelyants et al. 1993). It is also important to consider that deletion of genes involved in lysine biosynthesis may exert pleiotropic effects extending beyond the loss of the immediate metabolite. Disruption of the DAP pathway could alter the availability of key precursors required for cell wall synthesis and other regulatory processes, thereby indirectly influencing expression of genes involved in stress responses, membrane integrity, and virulence.

Investigating pathogenicity of the gene deletion mutants was achieved using the well‐established *G. mellonella* infection model (Benthall et al. 2015; Tsai et al. 2016). We sought to understand whether the generated gene deletion mutants were viable *in vivo* and still able to cause a bacterial infection. Interestingly, the Δ*dapB* mutant showed reduced pathogenicity in this model, meaning the larvae were able to survive over the 5‐day observation period and overcome the infection. This could indicate that there is no or not enough available *meso*‐DAP and/or lysine that can be scavenged to retain growth and allow the infection to proliferate. The Δ*lysA* mutant retained pathogenicity when injected into the larvae, indicating that sufficient concentrations of lysine are present in *Galleria* to sustain the infection and aid in growth of the bacteria. Previous reports indicate lysine concentrations in *G. mellonella* of 3.4 mM (Hanzal and Jegorov 1991), which is considerably higher than the minimum concentration required for successful Δ*lysA* growth as described in this study. Even though lysine supplementation can rescue growth of the Δ*dapB* mutant when cultured *in vitro*, this observation did not translate in this *in vivo* model. The lack of pathogenicity observed for the Δ*dapB* mutant would suggest that the strain cannot proliferate in this model even though sufficient lysine should be available to enable proliferation of the Δ*dapB* mutant infection. Furthermore, the mutation may cause a fitness penalty that affects virulence factors and membrane permeability and not bacterial growth, therefore impacting the ability of this strain to overcome the immune response of this model. While *G. mellonella* provides a cost‐effective and convenient model for initial studies, murine models offer advantages for investigating bacterial pathogenicity and virulence (Tsai et al. 2016). Mice possess a complex immune system that closely resembles human responses, enabling detailed analysis of bacterial interactions with host defenses (Sarkar and Heise 2019). In the case of the *dapB* and *lysA* mutants, it would be valuable to determine whether the concentrations of *meso*‐DAP and/or lysine available within a murine host are sufficient to maintain virulence or pathogenicity. Future complementation studies will also help confirm the role of the deleted genes and validate the phenotypes observed in the current work.

From these results, it was inferred that the membrane could be compromised, especially for the Δ*dapB* mutant, and this was first investigated utilizing the NPN assay. This assay helped elucidate differences in the membrane permeability of the mutant strains, with the Δ*dapB* mutant exhibiting an increase of 25.5% (DAP supplementation) and 39.3% (lysine supplementation) in outer membrane permeabilization when compared the WT strain. Interestingly, providing DAP to the culture did not recover membrane permeability back to WT levels, but was lower than mutants cultured in lysine. This finding supports the reasoning as to why the Δ*dapB* was unable to proliferate and infect the *G. mellonella* model. The Δ*lysA* mutant did not exhibit a large increase in membrane permeability, although it was significant. Observation of this was seen by TEM, with micrographs illustrating severe disruption to the outer membrane. *P. aeruginosa* is typically a rod‐shaped bacterium, however the Δ*dapB* mutant supplemented with lysine did not conform to the expected morphology, with non‐uniform shapes observed. This aligns with previous studies describing the importance of *meso*‐DAP to cell wall structure and shape (Wehrmann et al. 1998; Egan et al. 2017). This supports that *meso*‐DAP plays a pivotal role in providing stability to the cell membrane, and thus the overall shape of the *P. aeruginosa* cell. Future studies examining transporter expression could provide insights into potential compensatory mechanisms that maintain intracellular lysine levels in the mutant strains. Our findings led us to investigate whether current antibiotics could be more effective against these mutants.

Surveying the susceptibility of the mutant strains to existing antibiotics yielded exciting results. Ciprofloxacin and tobramycin have efficacy against and are used to treat, *Pseudomonas* infections (Fiel and Roesch 2022; Follath et al. 1986). When used against the Δ*dapB* and Δ*lysA* mutant strains, a decrease in MIC was observed. This increased efficacy could be supported by the observed increase in membrane permeability. One of the main challenges that antibiotics face is the complex and dynamic bacterial cell membrane of Gram‐negative bacteria, with many mechanisms including efflux pumps, employed to negate the effects of the drugs (Impey et al. 2020). Therefore, if the mutant strains exhibit defects that enhance the activity of current antibiotics, the associated enzymes of DHDPR and DAPDC could be potential targets for development of novel adjuvant therapeutics. Given that the DAP pathway is conserved among Gram‐negative bacteria, the phenotypes observed here may not be unique to *P*. aeruginosa, but could extend to other clinically relevant pathogens. This highlights the broader therapeutic potential of targeting enzymes such as DHDPR and DAPDC and supports further investigation of the DAP pathway as a promising avenue for antibiotic development.

Overall, here in this study, we illustrated the importance of *meso*‐DAP and lysine toward the growth, pathogenicity, and membrane permeability of *P. aeruginosa*. It is hoped this study can provide insight to help invigorate the antibiotic development pipeline to aid in the rising threat of antibiotic resistance.

## 4 Materials and Methods

### 4.1 Generation of Gene Deletion Mutants

Generation of *dapB* and *lysA* gene deletion mutants in *P. aeruginosa* (PAO1) was achieved using a previously optimized protocol (Hmelo et al. 2015; Huang and Wilks 2017). The 500 bp regions that flank upstream and downstream of each target gene were PCR amplified using specific primer sets (*dapB*; 1A & 2A, *lysA*; 1B & 2B) as described in Supplementary Table 1. The primer sets were designed to contain overhanging regions for subsequent cloning. Resulting PCR products were then cloned using NEB Hi‐Fi assembly master mix into the pEX18Tc plasmid ‘backbone’ that had been digested using EcoRI. The built construct was then transformed into *P. aeruginosa* PAO1 via electroporation as previously described (Huang and Wilks 2017). Successful transformants were seen on Luria‐Bertani (LB) agar plates containing 100 μg·mL^−^^1^ tetracycline grown at 37°C for up to 48 h. Colony PCR was performed to identify successful genome integration. This was achieved using One‐Taq PCR master mix (NEB) with specific primers described in Supplementary Table 1. Colonies that were shown to be integrated were re‐streaked onto tryptone, yeast, and sucrose (TYS) agar plates (10 g/L tryptone, 5 g/L yeast, and 10% (w/v) sucrose) supplemented with 1 mM DAP ( > 96% purity, Thermo Fisher Scientific) for Δ*dapB* mutants and 1 mM lysine (> 99% purity, Sigma Aldrich) for Δ*lysA* mutants. Growth on agar plates was achieved by incubation at room temperature (~ 22°C) for up to 72 h. Successful knockouts were screened for via colony PCR using primers (Supplementary Table 1). Gene deletion was further confirmed via Sanger sequencing performed by the Australian Genome Research Facility (Melbourne, VIC, Australia).

### 4.2 Growth Curve Analysis

A bacterial colony of WT PAO1, Δ*dapB* or Δ*lysA* was used to inoculate either supplemented (DAP or lysine) or non‐supplemented MOPS minimal media, prepared as previously described (Neidhardt et al. 1974). Subsequently, 1 mL cultures were incubated at 37°C with shaking at 160 rpm. After 1 h, 200 μL aliquots were added to clear 96‐well plates in technical triplicates and then placed in a FLUOStar Omega microplate reader (BMG Labtech). Plates were incubated at 37°C with shaking with change in absorbance readings taken at 600 nm (OD_600_) every 15 min for 30 h. Experiments were repeated with two biological replicates.

### 4.3 Galleria mellonella Infection Model

The *in vivo G. mellonella* assay was conducted as previously described (Ottonello et al. 2023). Bacteria from an overnight culture in tryptic soy broth of WT *P. aeruginosa*, Δ*dapB,* or Δ*lysA* were centrifuged with the pellet resuspended in 1 mL of PBS. Cultures were then diluted to an OD_600_ of 1 × 10^−^^6^ (2 CFU/10 μL) with 70 μL injected into a proleg of healthy *G. mellonella* (*N* = 10). Larvae were incubated statically at 37°C, with survival recorded daily for up to 5 days. Survival data were analyzed using the Kaplan‐Meier method, and statistical differences between survival curves were assessed using the log‐rank (Mantel‐Cox) test. A *p*‐value of < 0.05 was considered statistically significant.

### 4.4 Membrane Permeability Assays

Membrane permeability of the mutant strains compared to WT was assessed using the fluorometric NPN assay as previously described (Helander and Mattila‐Sandholm 2000; Hilton et al. 2023). In this study, overnight cultures of WT PAO1, Δ*dapB,* or Δ*lysA* were setup in MOPS minimal media containing either DAP or lysine supplementation at 1 mM and incubated at 37°C with shaking. Subsequently, subcultures were prepared at a ratio of 1:20 and grown at 37°C until an OD_600_ of 0.2 was reached to ensure mid‐logarithmic growth was achieved. Cultures were centrifuged and cell pellet were washed three times in 5 mM HEPES, 5 mM glucose, pH 7.2 buffer before a final resuspension in the same buffer. Assays were performed in black flat bottom 96‐well plates. Each well contained a final concentration of NPN of 10 μM. Assembled 96‐well plates were then placed in a FLUOStar Omega microplate reader (BMG Labtech) with excitation at 350 nm and emission at 420 nm. Assays were performed over a 20 min period with fluorescence intensity readings recorded every 1.5 min. Polymyxin B was used at 20 μg/mL as a positive control to ensure membranes could be permeabilized. NPN without the addition of bacterial culture was utilized as a negative control to account for any residual fluorescence. Fluorescence values obtained for each sample were first corrected by subtracting background fluorescence from NPN‐only wells. The resulting values were then normalized to the fluorescence of the positive control (polymyxin B‐treated cells), which was set to 100% permeability. Normalized data are expressed as a percentage of the maximal fluorescence response to enable direct comparison between strains. Experiments were carried out in three biological replicates.

### 4.5 TEM

For membrane visualization, TEM was employed. Cultures of either WT PAO1, Δ*dapB,* or Δ*lysA* were prepared with supplementation of either DAP or lysine at 1 mM. Cultures were subsequently pelleted at 2000 × g with supernatant carefully removed. Fixative containing 4% (w/v) sucrose, 1× PBS, 4% (v/v) paraformaldehyde, and 0.25% (v/v) glutaraldehyde was added, ensuring no disruption of the pellet, and left overnight. Fixative was then removed, and the pellet was washed three times with 1× PBS, with 2000 × g spins in between each wash step. Sample was then subjected to the addition of 1% (w/v) osmium tetroxide (OsO_4_) for 1 h before dehydrating in a 70%, 90%, 95%, and 100% (v/v) ethanol series. The ethanol was subsequently exchanged into 50% and then 100% (v/v) propylene oxide. Spurrs resin was prepared (ERL‐4221; 4.1 g, D.E.R. 736; 1.43, NSA; 5.9 g, DMAE; 0.1 g) and infiltrated in a 50:50 ratio with propylene oxide followed by two exchanges of 100% Spurrs resin (Spurr 1969). The sample resin mixture was then polymerized at 70°C for 48 h. Ultrathin sections of 70 nm were cut with a UC6 ultramicrotome (Leica, Macquarie Park, Australia) using a diamond knife (Diatome, Switzerland) and stretched by waving chloroform over the section. Sections were mounted on 100 mesh copper grids (ProScitech, Kirwan, Australia) and stained with 4% (v/v) uranyl acetate in distilled water followed by lead citrate (Graham and Orenstein 2007; Reynolds 1963; Nguyen et al. 2021). Sections were viewed using a Tecnai G2 Spirit (FEI Company, Hillsboro, OR, USA) transmission electron microscope at 100 kV. For this analysis, a single biological replicate was prepared, with two independently processed technical replicates, each comprising 4–6 ultra‐thin sections; representative micrographs are presented.

### 4.6 Antibacterial Assays

MIC values of the WT and gene deletion mutant strains were determined against two antibiotics (ciprofloxacin and tobramycin) using a broth microdilution method (Ottonello et al. 2023). Briefly, strains were maintained on MOPS minimal media agar plates containing 1 mM of supplementation (DAP or lysine). A subsequent overnight culture was prepared and then diluted to provide an inoculum of 1 × 10^5^ CFU/mL, which was added to MOPS minimal media in clear 96‐well plates. Supplementation in this assay was achieved via the addition of either DAP (Δ*dapB*; 1 mM) or lysine (Δ*dapB*; 1 mM, Δ*lysA*; 200 μM) to the appropriate wells. Growth was assessed after incubation at 37°C for 20 h by measuring the OD_600_ and compared to WT. Antibacterial assays were performed in three biological replicates.

## Author Contributions

**Daniel A. Hawkins:** data curation, formal analysis, writing – original draft, writing – review and editing; methodology. **Rachael E. Impey:** data curation, formal analysis, methodology, writing – review and editing. **Charlotte K. Hind:** data curation, formal analysis, methodology, writing – review and editing. **J. Mark Sutton:** formal analysis, methodology, resources, writing – review and editing. **Tatiana P. Soares da Costa:** conceptualization, funding acquisition, project administration, resources, writing – review and editing.

## Ethics Statement

The authos have nothing to report.

## Conflicts of Interest

None declared.

## Supporting information


**Supplementary Table 1.** Primers used for generation of *dapB* and *lysA* mutants.

## Data Availability

The data that support the findings of this study are available from the corresponding author upon reasonable request.
