# Supplementary material for: Probing the Role of Meso‐DAP and Lysine for Growth and Pathogenicity of Pseudomonas aeruginosa
Source: Microbiologyopen. 2025 Dec 22;14(6):e70200. doi: 10.1002/mbo3.70200 (PMC12723192; doi:10.1002/mbo3.70200)
Supplement: Supplementary file 1 — Supplementary Table 1. Primers used for generation of dapB and lysA mutants. [file MBO3-14-e70200-s001.docx]

**SUPPORTING INFORMATION**

**Supplementary Table 1.** Primers used for generation of *dapB* and *lysA* mutants.

| **Primer#** | **Sequence 5`-3`** | **Description** |
| --- | --- | --- |
| 1A & 2A | **1A:** ggaaacagctatgaccatgattacgGCCGTTGGCTTTCAGGTAGTCAGGC  **2A:** tggagtggttttGGCGCTGCCGCACGTCGTCTTATTG | 500 bp upstream and flanking region of the *dapB* gene, used for NEB Hi-Fi assembly* |
| 3A & 4A | 3A: gtgcggcagcgccAAAACCACTCCACGCGGAACCCGGAC  4A: agaggatccccgggtaccgagctcgCATCCGCCTCACCGGCGAAGGCGAG | 500 bp downstream flanking region of the *dapB* gene, used for NEB Hi-Fi assembly* |
| 1B & 2B | 1B: ggaaacagctatgaccatgattacgGTCGAGGTCCAGGTCGCTGTC  2B: atggtctgggccGGGCGCTCTCTCAGAAACCG | 500 bp upstream flanking region of the *lysA* gene, used for NEB Hi-Fi assembly* |
| 3B & 4B | 3B: tgagagagcgcccGGCCCAGACCATGCTTTTGC  4B: agaggatccccgggtaccgagctcgGCACGCCATGGGGATTGCCC | 500 bp downstream flanking region of the *lysA* gene, used for NEB Hi-Fi assembly* |
| 5 | 5: GGCTCGTATGTTGTGTGGAATTGTG | Universal pEX primer |
| 6A & 7A | 6A: GAGGATGCGAGTCAGGCGACG  7A: AGAAGACCCTGTCGGTGAAGGTGC | Upstream and downstream of the *dapB* gene, used for colony PCR and sequencing confirmation |
| 6B & 7B | 6B: TAATCGAAGTGGAAGCCGCCCG  7B: GAGGAAGCCGATGTTGGCCTTC | Upstream and downstream of the *lysA* gene, used for colony PCR and sequencing confirmation |

*For NEB Hi-Fi assembly reactions, lower case indicates homology regions used for cloning.
